# Locomotor, ecological and phylogenetic drivers of skeletal proportions in frogs

**DOI:** 10.1111/joa.13886

**Published:** 2023-05-19

**Authors:** Alice Leavey, Marcello Ruta, Christopher T. Richards, Laura B. Porro

**Affiliations:** ^1^ Centre for Integrative Anatomy, Cell and Developmental Biology University College London London; ^2^ Structure and Motion Laboratory Royal Veterinary College ‐ Camden Campus, Comparative Biomedical Sciences London; ^3^ Department of Life Sciences University of Lincoln, Joseph Banks Laboratories Lincolnshire United Kingdom

**Keywords:** 3D visualization, anuran, morphometrics, predictive analysis, ratio analysis

## Abstract

Frogs exhibit complex anatomical features of the pelvis, limbs and spine, long assumed to represent specialisations for jumping. Yet frogs employ a wide range of locomotor modes, with several taxa featuring primary locomotor modes other than jumping. Using a combination of techniques (CT imaging and 3D visualization, morphometrics, phylogenetic mapping), this study aims to determine the link between skeletal anatomy and locomotor style, habitat type and phylogenetic history, shedding new light on how functional demands impact morphology. Body and limb measurements for 164 taxa from all the recognised anuran families are extracted from digitally segmented CT scans of whole frog skeletons and analysed using various statistical techniques. We find that the expansion of the sacral diapophyses is the most important variable for predicting locomotor mode, which was more closely correlated with frog morphology than either habitat type or phylogenetic relationships. Predictive analyses suggest that skeletal morphology is a useful indicator of jumping but less so for other locomotor modes, suggesting that there is a wide range of anatomical solutions to performing locomotor styles such as swimming, burrowing or walking.

## Graphical abstract

This study investigates the link between skeletal anatomy and locomotor mode, habitat type and evolution in frogs, shedding new light on how functional demands impact morphology. Sacral expansion is the strongest driver of morphological variation. Locomotor mode has a more significant impact on morphology than habitat type and phylogenetic history. Skeletal morphology is a strong predictor of jumping, while there is a wide range of anatomical solutions to performing other locomotor modes.

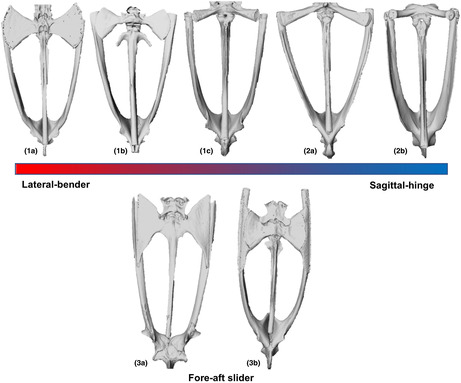

## 1 INTRODUCTION

The history of anuran morphometrics is dominated by a focus on the relationship between limb lengths and jumping performance (Rand, 1952; Zug, 1972; Dobrowolska, 1973; Choi et al., 2003; James et al., 2005; James et al., 2007; James & Wilson, 2008; Herrel et al., 2016). In contrast, other types of locomotor modes, including walking, hopping, swimming, burrowing and climbing, have received comparatively less attention (Emerson, 1979; Wells, 2007; Robovska‐Havelkova et al., 2014; Vassallo et al., 2021), as have regions of the body other than the pelvis and hindlimb such as the forelimb, hands and feet (Manzano et al., 2008, 2019; Keeffe & Blackburn, 2020, 2022; Abdala et al., 2022). Despite their relatively conserved body plan (Lires et al., 2016), extant anurans show considerable modifications in their skeletal proportions, reflecting their ability to respond to various mechanical challenges and inhabit diverse environments (Citadini et al., 2018; Gomes et al., 2009; Moen, 2019; Moen et al., 2013; Simons, 2008; Soliz et al., 2017; Vidal‐García et al., 2014). What remains unclear is how the interplay between morphology, function, ecology, and phylogeny drove the evolution of these modifications (Buttimer et al., 2020).

The total length of the hindlimb, and how it compares to forelimb and body length, is a good predictor of jumping performance, including jumping distance and take‐off speed (Choi et al., 2003; Emerson, 1978; Gomes et al., 2009; James & Wilson, 2008). While both terrestrial and arboreal jumpers have long hindlimbs, arboreal jumpers are said to have similarly elongated forelimbs to meet the biomechanical requirements for both climbing and jumping (Simons, 2008), or to compensate for the potential problem of a displaced centre of gravity (De Oliveira‐Lagôa et al., 2019). Similarly, more equally elongated forelimbs and hindlimbs have been shown to be associated with walking (Reynaga et al., 2018). However, comparatively fewer studies have considered whether individual hindlimb segments are associated with different functions during locomotion (Dobrowolska, 1973; Enriquez‐Urzelai et al., 2015; Lires et al., 2016). Additionally, the thickness of the forelimb relative to its length may potentially correlate with fossoriality, whereby proportionally larger humeral crests afford broader attachment sites for forelimb muscles used in digging (Emerson, 1976; Keeffe & Blackburn, 2020). Similarly, hindlimb thickness in aquatic species is associated with large muscles used for underwater propulsion (Gillis & Biewener, 2000). Significantly, however, a lack of detailed comparative investigations of limb ratios may be hampering conclusions about how locomotor function and skeletal proportions covary.

In addition to limb proportions, pelvic morphology has also been linked to variations in locomotor performance and habitat use (Emerson, 1979, 1982; Prikryl et al., 2009; Pugener & Maglia, 2009; Reilly & Jorgensen, 2011; Jorgensen & Reilly, 2013; Soliz et al., 2017; Buttimer et al., 2020). Pelvic specializations include the shape and degree of expansion of the sacral diapophyses (ESD), the presence or absence of dorsal ridges on the ilia and urostyle, and the morphology of the sacral‐urostylic joint. These features are widely used to identify three pelvic types, each associated with a specific type of movement, namely: ‘lateral‐bending’, ‘fore‐aft sliding’ and ‘sagittal‐hinge’. Previous studies have proposed that these pelvic types occur in walker‐hoppers, swimmers and jumpers, respectively (Emerson, 1979, 1982; Jorgensen & Reilly, 2013; Reilly & Jorgensen, 2011). More recently, it has been demonstrated that frog families (Manzano & Barg, 2005) and locomotor groups do not fall neatly into these groups (Simons, 2008; Soliz et al., 2017). Whether Emerson's three pelvic types accurately represent species‐level variation in pelvic morphology remains uncertain, suggesting that Emerson's concept of pelvic types should be revisited with a broader analysis.

Despite important progress made by previous studies, untangling the relationships between hindlimb/pelvic morphology and the habitats and locomotor modes of anurans remains challenging because of inconsistencies in the taxa examined, definitions of skeletal measurements, analytical methods and attribution of locomotor categories. For example, Buttimer et al. (2020) considered ‘burrowing’ to represent a habitat type rather than a locomotor mode. This attribution is particularly relevant as one of their major conclusions is that burrowing drove several morphological trends in anurans. As a result, direct comparisons between studies can be difficult and conclusive statements about the functional effects of different appendicular morphologies cannot be made with confidence.

To address the challenges above, the present study investigates the relationships between skeletal anatomy, locomotor mode and habitat type of anurans for 164 species spanning all extant frog families. We take detailed skeletal measurements using 3D visualisations of μCT scans to test the following hypotheses: (H1) hindlimb length/snout‐vent length ratio is highest in jumpers and lowest in fossorial taxa (Gomes et al., 2009; Vidal‐García et al., 2014); (H2) terrestrial jumpers have a higher hindlimb/forelimb length ratio, whereas this ratio approaches 1:1 in walker‐hoppers (Reynaga et al., 2018); (H3) hindlimb/forelimb length ratio is closer to 1:1 in arboreal jumpers than in terrestrial jumpers (Simons, 2008; De Oliveira‐Lagôa et al., 2019); (H4) different locomotor modes are correlated with differences in the relative lengths of individual hindlimb segments; specifically, the tibiofibular/femur length ratio is lower in swimmers and burrowers and higher in jumpers (Dobrowolska, 1973; Enriquez‐Urzelai et al., 2015); (H5) burrowers exhibit the widest, and, therefore, the most robust, forelimbs (Emerson, 1976; Keeffe & Blackburn, 2020), while aquatic species exhibit the most robust hindlimbs (Gillis & Biewener, 2000); (H6) narrow and wide ESD's predict terrestrial jumping and swimming, respectively (Emerson, 1979; 1982; Reilly & Jorgensen, 2011; Jorgensen & Reilly, 2013). We also investigate the extent to which phylogeny, locomotor mode and habitat type drive the evolution of frog morphology, as well as the ability of skeletal morphology to predict anuran lifestyle.

## 2 METHODS

### 2.1 Sampling

Skeletal measurements were taken from adult specimens of 164 anuran species representing all 54 families recognized on AmphibiaWeb (2021). We consider the families Pelodryadidae and Phyllomedusidae to be separate from Hylidae (Duellman et al., 2016), and Aromobatidae to be separate from Dendrobatidae (Grant et al., 2006). Our sampling size ranges from a single representative species for small families, such as Ascaphidae and Hemisotidae, to several species for large families, such as Hylidae and Microhylidae (Supplementary Dataset).

**FIGURE 1:**
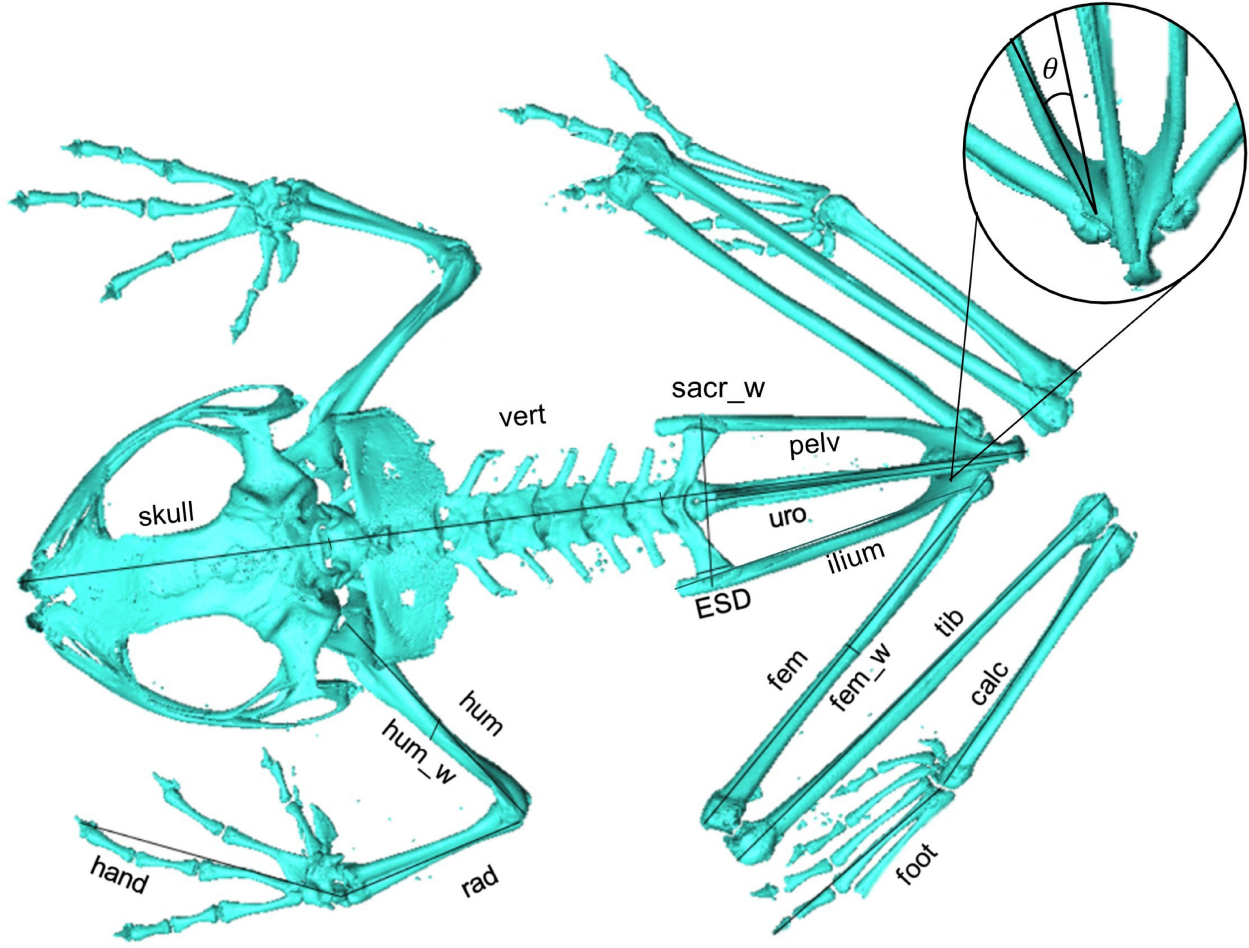
Morphometric measurements used in the analyses of the full dataset. [ESD] represents expansion of the sacral diapophysis and [_w] denotes width. For descriptive statistics and the structural dataset analysed in the SPCA, some measurements were combined to create total snout‐vent length, hindlimb length and forelimb length. See Table S1 for full measurement descriptions and Figure S1 for the iliac angle [θ] calculation.

We used the vast repository of recently acquired microcomputed tomography (μCT) scan data on MorphoSource.org (see ‘Full Dataset’ for ARK identifiers) to extract 22 skeletal measurements (Table S1) from each specimen using 3D measurement tools in Amira (Thermo Fisher Scientific). These include measurements of bones that have not been widely considered in previous studies, such as the calcaneus (tarsal segment) and various elements of the hand and foot. The width of the humerus and femur at midshaft were used as proxies for forelimb and hindlimb robusticity, respectively. Raw measurements were used to calculate iliac angle (Figure S1), total lengths for the body, hindlimb, foot, forelimb, and hand (Table S1), as well as 10 ratios which allow comparisons of relative lengths of individual limb segments, entire limbs, and body length as utilised in previous studies (Enriquez‐Urzelai et al., 2015; Petrović et al., 2017; De Oliveira‐Lagôa et al., 2019). Combination of selected raw measurements resulted in 16 morphological variables being analysed (Figure 1). For each specimen, all measurements were taken in dorsal view and from the left side. In the cases where bones of the left side were missing or incomplete, measurements were taken from the right side (19 out of 164 scans). In 23 specimens, the extremities of long bones were poorly ossified. Therefore, maximum length measurements in these specimens relied upon the ossified portions of each bone that could be detected in the scans. As the sex of most specimens was unknown, measurements were size‐corrected prior to analysis to mitigate the effects of dimorphism, given that females are larger than males in approximately 90% of frog species (Nali et al., 2014).

### 2.2 Pelvic morphology

We attempted to categorize taxa according to Emerson's (1979, 1982) pelvic types, using the shape of the sacral diapophysis (distally expanded ‘bow‐tie’/distally expanded flat edges/rod‐shape) and the absence/presence (half/full length of the bone) of dorsal crests on the iliac shaft and the urostyle. However, separation between pelvic types was not straightforward, particularly between sagittal‐hinge and lateral‐bending types, which appear to blend along a morphological continuum (see Discussion).

### 2.3 Phylogeny

To examine the impact of phylogeny on the evolution of skeletal structures, we trimmed the phylogenetic tree from Jetz and Pyron (2018) down to the species used in this study using R (Version 1.3.9, 2020) using the ‘keep. tip’ function in *ape* (Paradis & Schliep, 2019). The most recent nomenclature was utilised (IUCN, 2021) and three new species were added to the tree (Table S2) by replacing their most closely related congeneric taxon in the Jetz and Pyron (2018) tree to preserve the corresponding branch lengths, expressed in the form of substitutions per site. Three major phylogenetic groups—basal taxa (i.e., any species from before the evolution of the suborder Neobatrachia), Hyloidea and Ranoidea—were used as categories in our descriptive statistics and predictive analyses (see section 2.6). The Calyptocephalellidae, Myobatrachidae, Sooglossidae and Nasikabatrachidae are not within the superfamilies Hyloidea or Ranoidea, nor amongst the earliest evolving taxa (Jetz & Pyron, 2018), so the species from these families (*n* = 10) were grouped under their suborder ‘Neobatrachia’.

### 2.4 Locomotor modes

Information on locomotor mode was gathered from the literature (e.g., Jorgensen & Reilly, 2013; Keeffe & Blackburn, 2020) and through exchanges with researchers who have conducted first‐hand behavioural observations in the field (Andrew Gray and Dave Blackburn, pers. Comms.). We included aquatic swimmers (AQ), walker‐hoppers (WH), burrower‐walker‐hoppers (BWH) and both terrestrial jumpers (TJ) and arboreal jumpers (AJ) (Enriquez‐Urzelai et al., 2015). In line with previous literature, frogs categorised as jumpers can perform a leap greater than eight times their snout‐vent length, and choose to jump and hop more often than they walk (Emerson, 1979; Reilly et al., 2015; Soliz et al., 2017). Because the primary locomotor mode of 28 species is not known, the locomotor mode from closely related species from the same habitat was substituted. Assigning specific locomotor categories is sometimes challenging because many species use mixed locomotor styles depending on habitat type (Enriquez‐Urzelai et al., 2015). There were 20 cases where species had two observed locomotor modes (Supplementary Dataset). We assigned one primary locomotor mode per species in all cases. In uncertain cases, we considered a potential secondary locomotor mode by examining case‐wise predictive analyses (see section 2.6).

### 2.5 Habitat type

We assigned each species to one of four main habitat types: terrestrial, arboreal, riparian and aquatic (Gomes et al., 2009; Soliz et al., 2017). Riparian, or ‘semi‐aquatic’, describes frogs that spend comparable amounts of time in water and on land (Nauwelaerts et al., 2007). This information was collected from sources such as AmphibiaWeb and the IUCN website.

### 2.6 Statistical analyses

For each specimen, we adjusted the effect of size by dividing the measurements by their geometric mean. This isometric scaling results in dimensionless ratios also referred to as Mosimann shape variables (Mosimann, 1970). Previous studies have shown that these ratios perform better than residuals as size‐adjusted shape variables (Jungers et al., 1995). Furthermore, unlike residuals, Mosimann shape variables do not rely on trends from other individuals—they correct for scaling using information that relates solely to the specimen being measured (Sakamoto & Ruta, 2012). We carried out descriptive statistics to reveal morphological patterns and indicate which groups have more conserved anatomical features. The ratios specified in our hypotheses, as well as the means and standard error of each anatomical variable, were examined in relation to each locomotor mode, habitat type and major phylogenetic group (basal taxa, Hyloidea and Ranoidea). As ‘Neobatrachia’ only consists of 10 species from various positions within the phylogeny, it was excluded from descriptive statistics.

Subsequent statistical analyses were all carried out in R using log‐transformed Mosimann shape variables unless stated otherwise. We performed a phylogenetic principal component analysis (pPCA) under a Brownian motion model of evolution on the covariance matrix to reduce the dimensionality of the data and find the principal axes of variation (phyl.pca function in *phytools*; Revell, 2012). To test the significance of differences between groups, we ran a permutational multivariate analysis of variance (PERMANOVA) for locomotor mode, habitat type and phylogenetic group (*pairwiseAdonis*; Anderson, 2005). This analysis performs pairwise comparisons to test whether the means of various groups are similar.

Few studies have investigated the relationship between skeletal morphology and locomotor function using individual limb segments (but see Dobrowolska, 1973; Enriquez‐Urzelai et al., 2015; Lires et al., 2016; Gómez & Lires, 2019). To demonstrate the importance of analysing the length of each limb segment rather than only the larger structural features of frog morphology, we created a subset of the full dataset (Table 1), which combines some measurements to calculate total lengths for the body, hindlimb and forelimb (Table S1). Any variables that are not involved in these calculations remain unchanged. This is referred to as the ‘structural dataset’ (Table 1; Supplementary Information). Then, we performed two separate shape PCAs (SPCA; Baur & Leuenberger, 2011), one for each dataset. SPCA interprets a PCA in terms of ratios of body measurements by performing it in isometry free‐shape space and produces a PCA ratio spectrum which visualises the proportions that are most important when explaining the variance in each principal component (Baur & Leuenberger, 2011; Petrović et al., 2017). If individual hindlimb segments differ in explanatory power, then we have shown that they are important to consider in analyses of skeletal morphology compared to hindlimb length alone.

**TABLE 1:** Summary of the variables used in each dataset for the shape PCA analysis.

Measurement	Abbreviation	Full dataset	Structural dataset
Snout‐vent length	SVL		X
Skull	skull	X	
Vertebrae	vert	X	
Pelvis	pelv	X	
Sacral width	sacr_w	X	X
Ilium	ilium	X	X
Urostyle	uro	X	X
Iliac angle	θ	X	X
Hindlimb length	HL		X
Femur	fem	X	
Femur width	fem_w	X	X
Tibiofibula	tib	X	
Calcaneus	calc	X	
Foot	foot	X	
Forelimb length	FL		X
Humerus	hum	X	
Humerus width	hum_w	X	X
Radioulna	rad	X	
Hand	hand	X	

Using *nlme* (Pinheiro, 2012), *ape* (Paradis & Schliep, 2019), and the species' scores from PC1 and PC2 as the dependent variables, we carried out a phylogenetic least squares (PGLS) analysis to determine the extent to which variation in skeletal morphology is driven by phylogeny, locomotor mode and habitat type. The phylogenetic signal was extracted using Pagel's lambda (λ; Pagel, 1999). PC1 and PC2 scores were mapped onto the phylogeny using *RcolorBrewer* (Neuwirth & Neuwirth, 2011) and the ‘contMap’ function in *phytools* (Revell, 2012).

We also investigated how well skeletal morphology predicts the attribution of each species to its locomotor mode, habitat type and phylogenetic group categories. To evaluate the predictive power of our measurement data, we performed two types of analyses: linear discriminant analyses (LDA; lda function, MASS package) and phylogenetic flexible discriminant analysis (pFDA; phylo.fda function; Motani & Schmitz, 2011). Both seek to establish whether the measurement data are able to retrieve the same categories or, with regard to taxa with debatable primary locomotor modes, the alternative locomotor mode. Misclassifications indicate that the morphology of that species falls outside the range estimated for that locomotor mode, habitat type or phylogenetic group based on the data provided. The data input for the phylogenetic group analyses did not include species from the ‘Neobatrachia’ group, but we utilised the LDA and pFDA (phylo.fda.pred function; Motani & Schmitz, 2011) to predict which phylogenetic group these species would be allocated to, given their morphology. For the pFDA, the optimal Pagel's lambda was used, which maximises the correlation between locomotor mode/habitat type and the morphological variables (Motani & Schmitz, 2011). The rationale behind using both analyses was that we were interested in ascertaining the predictive power of morphological variables with (pFDA) and without (LDA) accounting for phylogenetic covariance.

## 3 RESULTS

### 3.1 Skeletal morphology grouped by locomotor mode, habitat type and phylogenetic group

**FIGURE 2:**
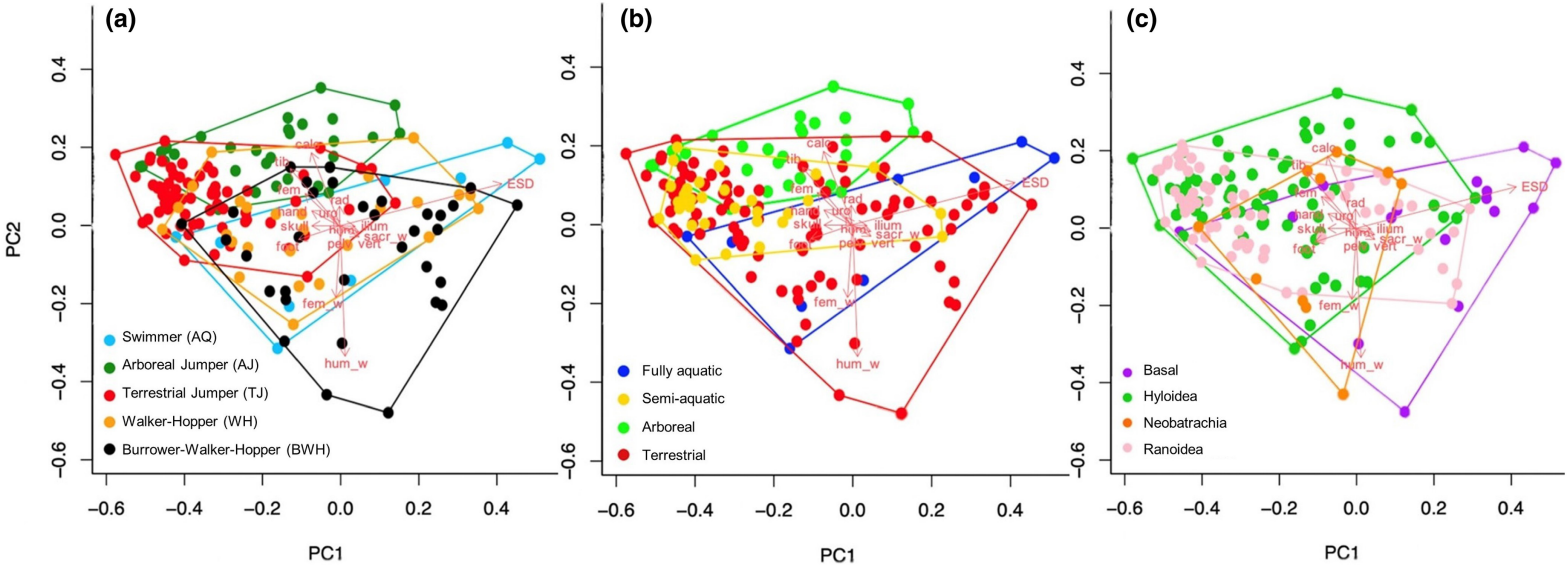
pPCA of morphometric measurements from the full dataset coloured‐coded according to three alternative groupings of locomotor mode (a), habitat type (b) and phylogenetic group (c). The red arrows represent the pPCA loadings.

The results of our descriptive statistics for the size‐corrected measurements and key ratios can be found in Table 2. The first two principal components (PCs) from the pPCA explain 34.2% and 21.9% of the total variance in the data, respectively (Table S3). Five PC axes are required to explain ~80% of the total variance. The morphological space occupied by each species can be defined according to their position on the first PC axis – species with larger sacral expansions have higher PC1 scores (Table S3; Figure 2). Higher scores on the second PC axis indicate species with a long tibiofibula and calcaneus, and the lowest scores correspond to species with large humeral ridges. In terms of morphospace occupations, all groups appear to overlap considerably. PERMANOVA tests indicate that skeletal morphology differs significantly across locomotor mode, habitat type and phylogenetic group (Table S4). Specifically, in terms of locomotor mode, AJ and TJ are significantly separated from each other, as well as from AQ, BWH and WH, which show a wider occupancy of morphospace. There is no significant separation between AQ, BWH and WH. For habitat type, arboreal taxa are significantly separate from all other taxa, and riparian and aquatic taxa are significantly separated from each other. There is no significant separation between terrestrial taxa and each of the riparian and aquatic taxa. When arranged by phylogenetic group, Hyloidea and Ranoidea were not significantly different from each other, but the basal taxa were distinct from both of the more derived groups.

**TABLE 2:** Descriptive statistics of the key morphometric measurements and ratios discussed in this study for locomotor mode, habitat type and major phylogenetic group. Light and dark boxes indicate the highest and lowest values respectively. Measurement abbreviations can be found in Table 1. The values are displayed as the mean ± standard error, and the number in brackets indicates the number of species in that group. The iliac angle is a raw measurement, whereas all other anatomical measurements shown have been corrected for size (see section 2.6) but not log‐transformed for ease of interpretation. The full dataset can be obtained in the supplementary information.

Measurement	Locomotor mode					Habitat				Phylogenetic group		
	BWH (33)	WH (26)	TJ (66)	AJ (30)	AQ (9)	Terrestrial (96)	Riparian (31)	Arboreal (28)	Aquatic (9)	Basal (16)	Hyloidea (72)	Ranoidea (65)
**SVL (cm)**	5.930 ± 0.478	5.646 ± 0.669	4.944 ± 0.370	6.310 ± 0.470	7.265 ± 0.975	5.003 ± 0.278	6.470 ± 0.662	6.329 ± 0.481	7.265 ± 0.975	6.829 ± 0.601	5.362 ± 0.336	5.491 ± 0.387
**ESD (cm)**	2.291 ± 0.185	2.141 ± 0.264	1.856 ± 0.148	2.382 ± 0.191	2.815 ± 0.377	1.898 ± 0.107	2.461 ± 0.276	2.382 ± 0.195	2.815 ± 0.377	2.667 ± 0.268	2.010 ± 0.131	2.072 ± 0.149
**sacr_w (cm)**	0.414 ± 0.049	0.357 ± 0.064	0.161 ± 0.014	0.310 ± 0.036	0.665 ± 0.168	0.275 ± 0.026	0.247 ± 0.042	0.315 ± 0.038	0.665 ± 0.168	0.796 ± 0.118	0.266 ± 0.023	0.209 ± 0.018
**HL (cm)**	7.384 ± 0.720	7.164 ± 0.780	7.505 ± 0.604	9.673 ± 0.777	8.771 ± 0.959	6.653 ± 0.376	9.913 ± 1.073	9.624 ± 0.765	8.771 ± 0.959	8.566 ± 0.685	7.656 ± 0.483	7.788 ± 0.603
**foot (cm)**	2.206 ± 0.200	2.063 ± 0.230	2.120 ± 0.171	2.284 ± 0.179	2.742 ± 0.320	1.922 ± 0.108	2.800 ± 0.300	2.268 ± 0.175	2.742 ± 0.320	2.486 ± 0.191	2.068 ± 0.125	2.188 ± 0.170
**FL (cm)**	4.097 ± 0.333	4.174 ± 0.493	3.549 ± 0.261	4.629 ± 0.342	4.870 ± 0.786	3.562 ± 0.202	4.683 ± 0.449	4.633 ± 0.347	4.870 ± 0.786	4.708 ± 0.380	3.971 ± 0.257	3.852 ± 0.268
**hand (cm)**	1.366 ± 0.109	1.419 ± 0.172	3.549 ± 0.261	1.805 ± 0.141	1.696 ± 0.274	1.212 ± 0.070	1.663 ± 0.158	1.810 ± 0.144	1.696 ± 0.274	1.601 ± 0.122	1.441 ± 0.099	1.350 ± 0.268
**Iliac angle (°)**	8.865 ± 0.403	9.924 ± 0.548	7.952 ± 0.209	9.474 ± 0.394	8.574 ± 0.794	8.593 ± 0.229	8.690 ± 0.471	9.476 ± 0.414	8.574 ± 0.794	8.664 ± 0.653	9.317 ± 0.285	8.159 ± 0.255
**Ratio**												
**HL/FL**	1.807 ± 0.062	1.748 ± 0.044	2.105 ± 0.035	2.071 ± 0.031	1.946 ± 0.132	1.917 ± 0.035	2.071 ± 0.052	2.070 ± 0.028	1.946 ± 0.132	1.848 ± 0.074	1.962 ± 0.037	2.010 ± 0.039
**HL/SVL**	1.224 ± 0.038	1.290 ± 0.037	1.507 ± 0.023	1.526 ± 0.032	1.249 ± 0.050	1.351 ± 0.024	1.510 ± 0.034	1.523 ± 0.031	1.249 ± 0.050	1.275 ± 0.047	1.440 ± 0.027	1.405 ± 0.028
**ESD/HL**	0.061 ± 0.006	0.048 ± 0.006	0.023 ± 0.001	0.033 ± 0.003	0.075 ± 0.019	0.042 ± 0.003	0.027 ± 0.003	0.033 ± 0.003	0.075 ± 0.019	0.092 ± 0.012	0.035 ± 0.002	0.031 ± 0.003
**fem/HL**	0.276 ± 0.003	0.269 ± 0.004	0.272 ± 0.004	0.281 ± 0.002	0.285 ± 0.011	0.274 ± 0.003	0.267 ± 0.003	0.281 ± 0.002	0.285 ± 0.011	0.272 ± 0.005	0.277 ± 0.004	0.274 ± 0.002
**tib/HL**	0.272 ± 0.004	0.282 ± 0.003	0.299 ± 0.004	0.304 ± 0.002	0.273 ± 0.006	0.287 ± 0.003	0.293 ± 0.003	0.305 ± 0.002	0.273 ± 0.006	0.277 ± 0.005	0.296 ± 0.004	0.289 ± 0.002
**calc/HL**	0.151 ± 0.003	0.163 ± 0.004	0.177 ± 0.003	0.177 ± 0.003	0.155 ± 0.008	0.159 ± 0.003	0.156 ± 0.003	0.177 ± 0.003	0.155 ± 0.008	0.165 ± 0.004	0.169 ± 0.003	0.149 ± 0.006
**foot/HL**	0.302 ± 0.006	0.285 ± 0.007	0.282 ± 0.005	0.237 ± 0.003	0.313 ± 0.015	0.288 ± 0.004	0.285 ± 0.006	0.237 ± 0.003	0.313 ± 0.023	0.293 ± 0.009	0.274 ± 0.006	0.282 ± 0.004
**tib/fem**	0.989 ± 0.017	1.055 ± 0.018	1.101 ± 0.009	1.084 ± 0.008	0.994 ± 0.021	1.050 ± 0.010	1.102 ± 0.015	1.083 ± 0.009	0.994 ± 0.021	1.025 ± 0.034	1.076 ± 0.009	1.061 ± 0.010
**fem_w/fem**	0.074 ± 0.004	0.064 ± 0.002	0.053 ± 0.001	0.044 ± 0.001	0.074 ± 0.006	0.062 ± 0.002	0.055 ± 0.002	0.045 ± 0.001	0.074 ± 0.006	0.071 ± 0.006	0.055 ± 0.002	0.057 ± 0.002
**hum_w/hum**	0.132 ± 0.009	0.110 ± 0.004	0.096 ± 0.003	0.130 ± 0.012	0.130 ± 0.023	0.111 ± 0.004	0.098 ± 0.003	0.089 ± 0.002	0.130 ± 0.023	0.142 ± 0.012	0.103 ± 0.004	0.097 ± 0.003

### 3.2 Shape PCA spectrums for visualising the relative importance of structural morphological ratios

**FIGURE 3:**
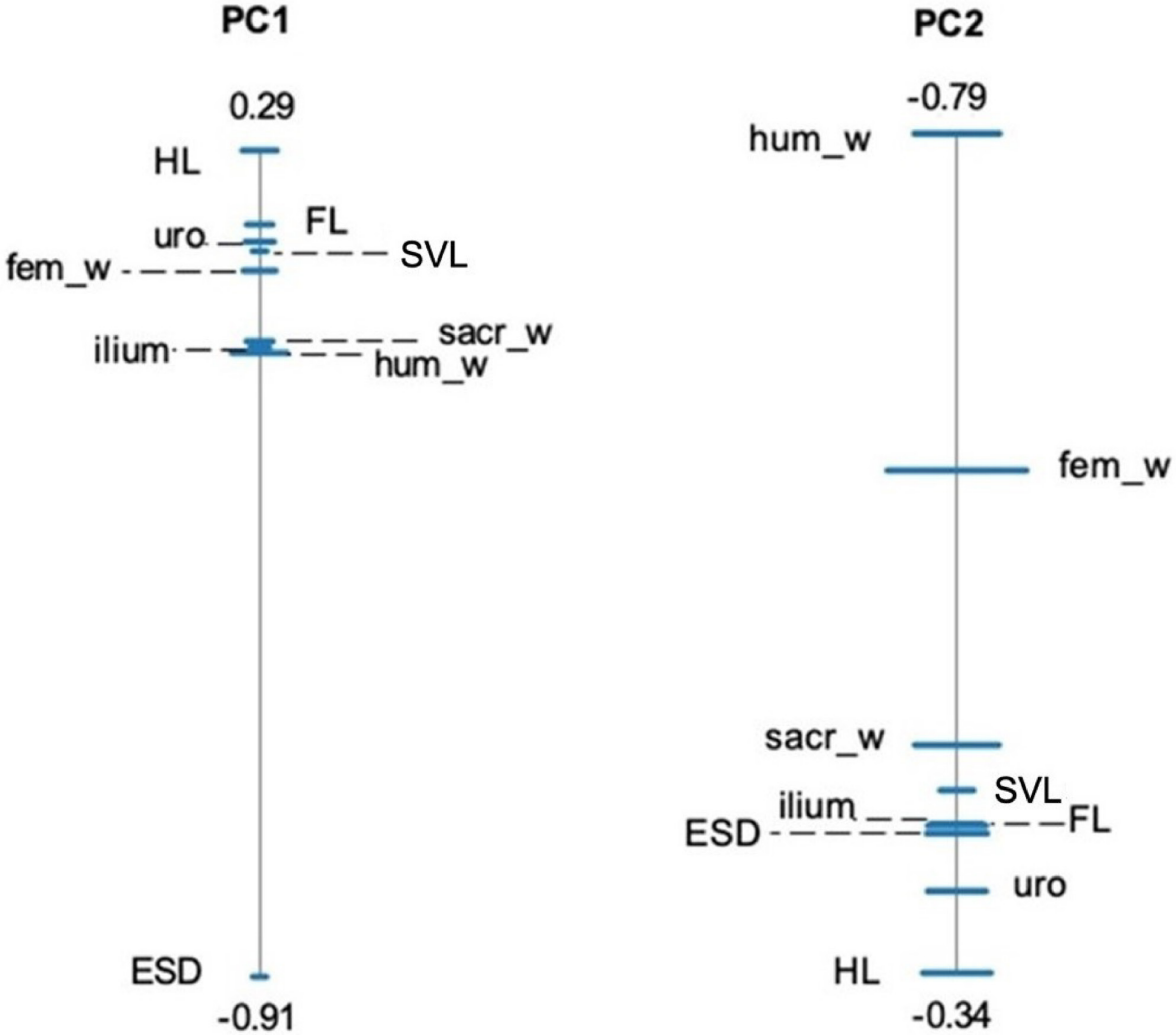
Shape PCA ratio spectra for PC1 and PC2, which shows the most dominant ratios and their interrelationships (Baur & Leuenberger, 2011; Petrović et al., 2017) for our structural data subset containing total body and limb lengths. A SPCA spectrum visualises the proportions that are most important when explaining the variance in each principal component. Bars represent 68% confidence intervals based on 999 bootstrap replicates. Variable labels alternate from left to right; dashed lines are used to distinguish between those that are very closely positioned. Variables positioned close to each other depict ratios that explain little variation, whereas those at the opposite ends of each spectrum represent a ratio with high explanatory power. In this case, the ratio of hindlimb length/snout‐vent length and humerus width/hindlimb length have the highest explanatory power for PC1 and PC2, respectively. The numbers at each end of the spectrum represent the highest and lowest PC loadings of the two most opposite variables. See Table 1 for abbreviations.

**FIGURE 4:**
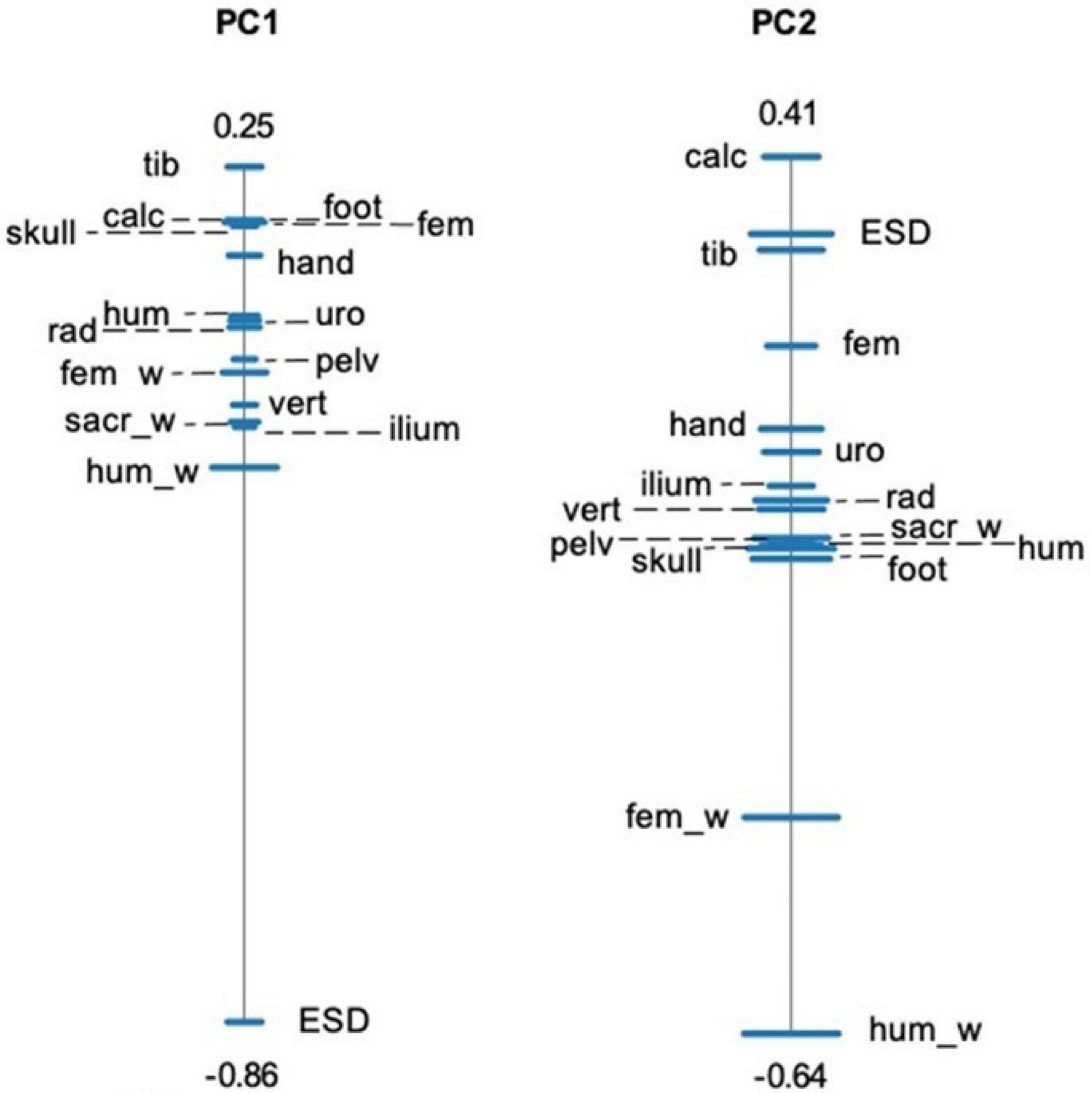
Shape PCA ratio spectra for PC1 and PC2, which shows the most dominant ratios and their interrelationships (Baur & Leuenberger, 2011; Petrović et al., 2017) for our full dataset. A SPCA spectrum visualises the proportions that are most important when explaining the variance in each principal component. Bars represent 68% confidence intervals based on 999 bootstrap replicates. Variable labels alternate from left to right; dashed lines are used to distinguish between those that are very closely positioned. Variables positioned close to each other depict ratios that explain little variation, whereas those at the opposite ends of each spectrum represent a ratio with high explanatory power. In this case, the ratio of tibiofibula length/sacral expansion and calcaneus length/humerus width have the highest explanatory power for PC1 and PC2, respectively. The numbers at each end of the spectrum represent the highest and lowest PC loadings of the two most opposite variables. See Table 2 for abbreviations.

For our SPCA analysing the structural dataset, the first two principal components (PCs) explain 67.1% and 16.5% of the total variance in our subset of nine variables. Most of the variation in shape PC1 is explained by the hindlimb length/ESD ratio (Table S5), which corresponds to the position of these two variables at the opposite ends of the PC1 ratio spectrum (Figure 3). The humerus width/hindlimb length ratio drives variation in shape PC2 (Table S5; Figure 3). In our full dataset, shape PC1 and PC2 explain, respectively, 56.7% and 16% of variance. Tibiofibula/ESD ratio explains most of the variation in shape PC1, while humerus width/calcaneus length is the most dominant ratio driving shape PC2 (Table S6; Figure 4). However, note that the PC2 ratio spectrums for both datasets have wider error bars, which occurs when PC values are less significantly separated from each other. Therefore, the wider error bars indicate that less definitive conclusions can be made from PC2 (Baur & Leuenberger, 2011). Allometry ratio spectrums show that shape was not significantly correlated with size for both datasets (Figure S2).

### 3.3 Phylogenetic analyses

**FIGURE 5:**
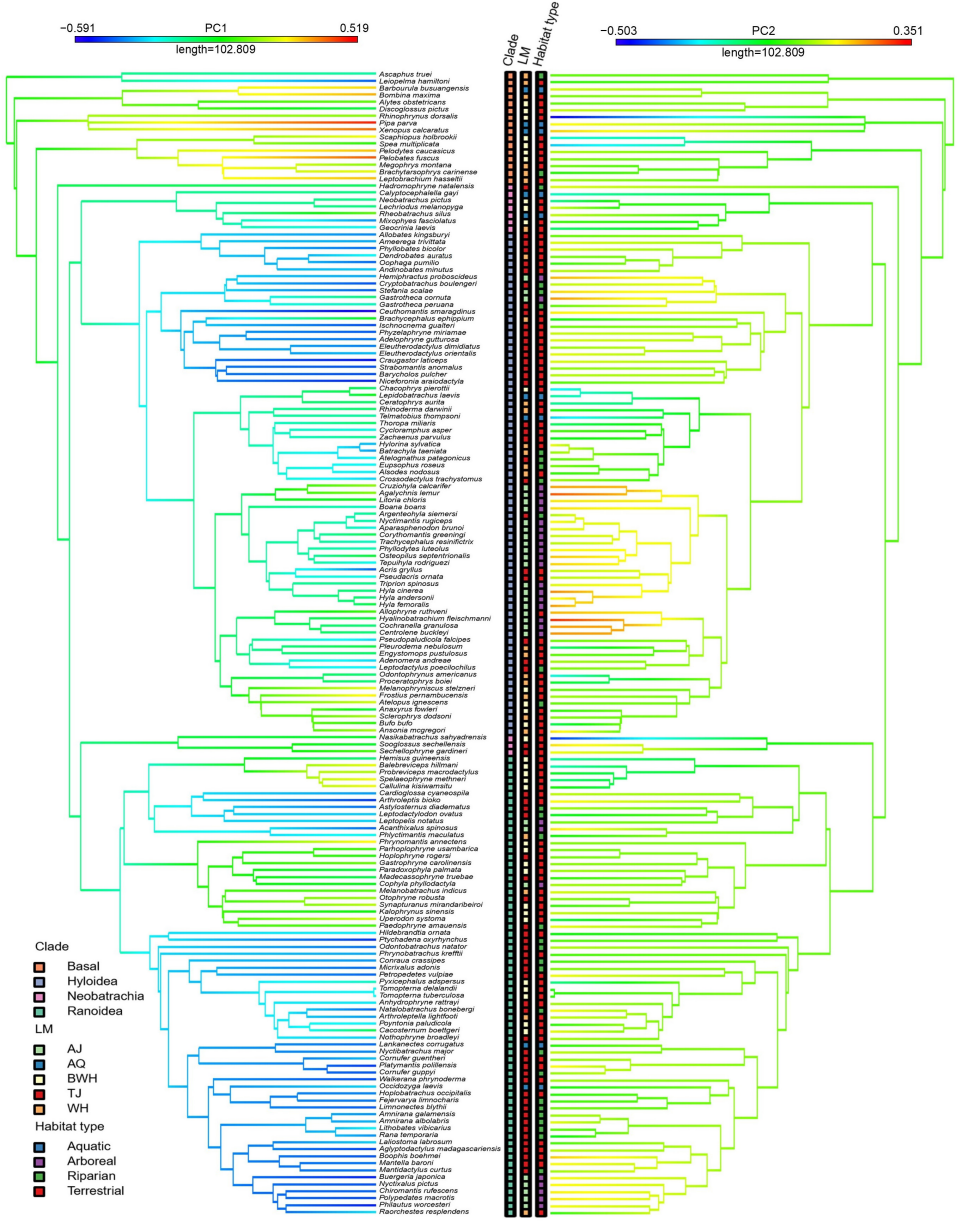
Our phylogeny of 164 frog species based on Jetz & Pryon (2018). Warmer tip colours (red, yellow) represent more positive scores for PC1 (left) and PC2 (right) from the full dataset. Tips are labelled with a colour‐coded grid to represent the categorical variables investigated (see text for abbreviations). The 10 species outside of the Hyloidea and Ranoidea superfamilies have been referred to as ‘Neobatrachia’.

By plotting the scores obtained from the pPCA onto the phylogeny, we are able to visualise the evolution of skeletal morphology (Figure 5). The best PGLS models involved both locomotor mode and habitat type for PC1, but only locomotor mode for PC2 (Table S7). The most significant predictor of skeletal morphology was locomotor mode in both analyses (Table 3). Phylogenetic signal was greater than one for analyses of PC1, indicating the signal is stronger near the root of the phylogeny compared to the tips (Pagel, 1999), while the phylogenetic signal was weaker for PC2 (Table S7).

**TABLE 3:** Coefficients from the best PGLS models describing the relationship between variation in skeletal morphology (PC1 scores and PC2 scores from the full dataset) and locomotor mode (LM) and habitat type for 164 frog species. SEM = standard error. Significant results are highlighted in bold.

Model		Coefficients	SEM	*t*‐value	*p*‐value
PC1 ~ LM + Habitat	Intercept	−0.056	0.08	−0.701	0.484
	LM	0.049	0.013	3.892	**<0.001**
	Habitat	−0.03	0.014	−2.081	**0.039**
PC2 ~ LM	Intercept	0.141	0.042	3.392	**<0.001**
	LM	−0.053	0.008	−7.03	**<0.001**

### 3.4 The predictive power of skeletal morphology

The classification of each species into locomotor mode, habitat type and phylogenetic group based on skeletal morphology across both types of discriminant analysis is available in the ‘predictive analyses dataset’ (see supplementary information). For the LDA, when taxa are grouped by locomotor mode, we find that LD1 and LD2 explain, respectively, 61.5% and 26.3% of the data variance. pFDA1 and pFDA2 explained 44.6% and 26.6% of data variance. Overall, LDAs assigned 77.4% of species to the correct locomotor mode, while the pFDA correctly assigned 62.8%. AJs (LDA: 96.7%; pFDA: 63.3%) and TJs (LDA: 90.9%; pFDA: 77.3%) are correctly classified most frequently, and AJs are only ever misclassified as TJs in the LDA (3.3%), indicating that the morphology in these frogs is more characteristic of their locomotor modes (Tables 4 and 5). WH, BWH and AQ are predicted correctly less frequently, suggesting these locomotor modes are less constrained by their morphology.

**TABLE 4:** Classification results from the linear discriminant analysis (LDA) of the full dataset for locomotor mode, habitat type and phylogenetic group. Neobatrachians consist of 10 species was not used as inputs for the predictive model.

	Locomotor mode	Predicted group membership – 77.4% accuracy					Total
		WH	BWH	TJ	AJ	AQ	
Count	WH	14	6	5	0	1	26
	BWH	6	20	6	0	1	33
	TJ	3	1	60	2	0	66
	AJ	0	0	1	29	0	30
	AQ	1	2	2	0	4	9
%	WH	53.8	23.1	19.2	0.0	3.8	100
	BWH	18.2	60.6	18.2	0.0	3.0	100
	TJ	4.5	1.5	90.9	3.0	0.0	100
	AJ	0.0	0.0	3.3	96.7	0.0	100
	AQ	11.1	22.2	22.2	0.0	44.4	100

	Predicted group membership – 76.8% accuracy				
	Habitat type	Terrestrial	Riparian	Arboreal	Aquatic	Total	
Count	Terrestrial	85	5	4	2	96	
	Riparian	18	11	2	0	31	
	Arboreal	1	1	26	0	28	
	Aquatic	3	2	0	4	9	
%	Terrestrial	88.5	5.2	4.2	2.1	100	
	Riparian	58.1	35.5	6.5	0.0	100	
	Arboreal	3.6	3.6	92.9	0.0	100	
	Aquatic	33.3	22.2	0.0	44.4	100	

	Phylogenetic group	Predicted Group Membership – 75.3% accuracy (excl. Neobatrachia)			Total		
		Basal	Hyloidea	Ranoidea			
Count	Basal	11	5	0	16		
	Hyloidea	0	54	18	72		
	Ranoidea	1	15	50	66		
	Neobatrachia	1	6	3	10		
%	Basal	68.8	31.3	0.0	100		
	Hyloidea	0.0	75.0	25.0	100		
	Ranoidea	1.5	22.7	75.8	100		
	Neobatrachia	10.0	60.0	30.0	100		

**TABLE 5:** Classification results from the phylogenetic flexible discriminant analysis (pFDA) of the full dataset for locomotor mode, habitat type and phylogenetic group. Neobatrachians consist of 10 species was not used as inputs for the predictive model.

	Locomotor mode	Predicted group membership – 62.8% accuracy					Total
		WH	BWH	TJ	AJ	AQ	
Count	WH	12	5	8	0	1	26
	BWH	6	16	6	1	4	33
	TJ	6	6	51	3	0	66
	AJ	1	4	5	19	1	30
	AQ	1	3	0	0	5	9
%	WH	46.2	19.2	30.8	0.0	3.8	100
	BWH	18.2	48.5	18.2	3.0	12.1	100
	TJ	9.1	9.1	77.3	4.5	0.0	100
	AJ	3.3	13.3	16.7	63.3	3.3	100
	AQ	11.1	33.3	0.0	0.0	55.6	100

	Habitat type	Predicted group membership – 65.2% accuracy				Total	
		Terrestrial	Riparian	Arboreal	Aquatic		
Count	Terrestrial	80	7	2	7	96	
	Riparian	20	11	0	0	31	
	Arboreal	15	0	12	1	28	
	Aquatic	5	0	0	4	9	
%	Terrestrial	83.3	7.3	2.1	7.3	100	
	Riparian	64.5	35.5	0.0	0.0	100	
	Arboreal	53.6	0.0	42.9	3.6	100	
	Aquatic	55.6	0.0	0.0	44.4	100	

	Phylogenetic group	Predicted Group Membership – 71.4% accuracy (excl. Neobatrachia)			Total		
		Basal	Hyloidea	Ranoidea			
Count	Basal	11	5	0	16		
	Hyloidea	2	52	18	72		
	Ranoidea	0	19	47	66		
	Neobatrachia	10	0	0	10		
%	Basal	68.8	31.3	0.0	100		
	Hyloidea	2.8	72.2	25.0	100		
	Ranoidea	0.0	28.8	71.2	100		
	Neobatrachia	100.0	0.0	0.0	100		

Based on the literature, locomotor mode was uncertain for 22 taxa (see section 2.4). We examined the case‐wise statistics of our predictive analyses to see if the alternative locomotor mode was correctly predicted. In the LDA and pFDA, respectively, 14 and 10 species had their primary locomotor mode predicted correctly, while eight and nine species were predicted to have the alternative locomotor mode, supporting the predictive power of the model. Additionally, where the locomotor mode from a closely related proxy species was used, the LDA and pFDA correctly predicted the locomotor mode for 23 and 15 out of the 28 species, respectively. These results highlight why entire families should not be grouped under a single locomotor type, as this could vary at genus or species‐level. Additionally, where we were certain of locomotor mode, the pFDA made more incorrect classifications (48) than the LDA (28), suggesting that the inclusion of phylogenetic history weakens the predictive power of skeletal morphology.

For habitat type, LD1 and LD2 explain, respectively, 79% and 12.4% of the variance in the data. pFDA1 and pFDA2 explain 60.2% and 23.6%. Classification was successful in 76.8% of species in the LDA (Table 4) and 65.2% in the pFDA (Table 5). For the LDA, arboreal (92.9%) and terrestrial (88.5%) taxa are classified correctly most often, but riparian species are frequently misclassified as terrestrial (58.1%). The same conclusion holds true for the pFDA, except that arboreal species were often mistaken for being terrestrial (53.6%). In the 17 cases of habitat type uncertainty, the primary habitat type was predicted correctly for seven species in the LDA and six species in the pFDA, and the potential alternative habitat type was predicted in six and four species, respectively. There were 28 (LDA) and 45 (pFDA) cases where habitat type was incorrectly classified despite certainty.

When grouped by phylogenetic group excluding the ‘Neobatrachia’, LD1 and LD2 explain 78.3% and 21.7% of the variance in the data, respectively. pFDA1 and pFDA2 explain 85.1% and 14.9%. Correct classifications were almost equal across the groups in the LDA, with 75.3% of species correctly categorised overall (Table 4). For the pFDA, Ranoidea and Hyloidea were correctly classified most often, with an overall accuracy of 71.4% (Table 5). The ten species in the Neobatrachia group were mainly categorised as Hyloidea and Ranoidea, with *Calyptocephalella gayi* classified as basal in the LDA, while the pFDA suggested that all neobatrachians belong in the basal group based upon their skeletal morphology.

## 4 DISCUSSION

This study uses a combination of newly acquired μCT data, comparative morphometrics and phylogenetic comparative methods to comprehensively measure and analyse the hindlimb and pelvis anatomy of a broad range of anurans. We have uncovered several important correlations between anuran skeletal proportions and locomotor mode, habitat type and phylogeny. In summary, the impact of locomotor function on the evolution of frog anatomy is reflected in the different functional roles of individual limb segments. Some locomotor modes and habitat types are associated with less conserved skeletal morphologies than others, suggesting multiple anatomical solutions for achieving the same function. Additionally, we found that pelvic morphology, a key predictor of locomotor mode in anurans, forms a continuum, thereby rendering Emersonian morphotypes as unreliable for identifying species and predicting locomotor mode and habitat type. Additionally, by testing two types of predictive analyses using extant taxa, we demonstrate how skeletal morphology could be used to predict the lifestyle of extinct species in future studies.

### 4.1 Body and limb proportions show distinct patterns in relation to locomotor function

In agreement with recent findings and with H1, hindlimb length/snout‐vent length values are highest in arboreal jumpers (AJ) and decrease across terrestrial jumpers (TJ), walker‐hoppers (WH), swimmers (AQ) and burrower‐walker‐hoppers (BWH), suggesting that proportionately longer hindlimbs enhance jumping performance (Choi et al., 2003; Gomes et al., 2009; Herrel et al., 2016; James & Wilson, 2008; Vidal‐García et al., 2014). On average, TJ hindlimbs are 2.1x longer than their forelimbs, while WH have a hindlimb/forelimb length ratio closer to 1:1, in agreement with H2. AJ were also expected to have a hindlimb/forelimb ratio closer to 1:1, as AJ were previously thought to be constrained by a functional trade‐off due to jumping versus climbing demands (Simons, 2008; Enriquez‐Urzelai et al., 2015; De Oliveira‐Lagôa et al., 2019). In contradiction to H3, AJ have the second highest hindlimb/forelimb length ratio. This suggests that while the optimal forelimb length for climbing is lower than the optimal forelimb length for jumping, both sets of limbs do not need to be equally elongated to enable tree frogs to reach distant branches. This is potentially because jumping may be used as an important escape mechanism or the primary mode of locomotion. Alternatively, it is possible that the measurements included in our study may not capture morphology particularly adapted for climbing locomotion (e.g., toes pads).

As expected, the relative lengths of different hindlimb segments vary across locomotor modes and habitats (H4). Hindlimb elongation in AQ and BWH frogs occurs primarily in the femur, in agreement with previous studies (Enriquez‐Urzelai et al., 2015; Lires et al., 2016). The lengths of the tibiofibula and calcaneus drive hindlimb elongation in AJ and TJ (Table 2), as found in previous studies (James & Wilson, 2008; Jorgensen & Reilly, 2013; Enriquez‐Urzelai et al., 2015; Lires et al., 2016; Gómez & Lires, 2019). Support for H5 was less clear. BWH are characterized by the most robust forelimbs and hindlimbs, thus supporting large muscles involved in forward‐ and backward‐burrowing (Emerson, 1976; Keeffe & Blackburn, 2020). In terms of habitat type, aquatic taxa have the most robust hindlimb and forelimb. However, when interpreting any trends in habitat type, it is important to note that terrestrial taxa are comprised of a mix of TJ, BWH and WH, which have contrasting morphologies. Terrestrial taxa had relatively average means for most anatomical variables, indicating that some effects are being cancelled out. This emphasises the importance of considering more than just broad habitat types when investigating correlations between morphology, function, and ecology, as well as highlighting the need for more quantitative ecological data.

AJ and TJ exhibit the least variation across all ratios, indicating that the skeletal proportions of jumping frogs are conserved. In contrast, AQ are the most variable, suggesting there may be more anatomical solutions to achieve satisfactory swimming performance. This supports findings by previous studies analysing swimming across different frog species (Richards, 2010; Robovska‐Havelkova et al., 2014) found differences in pelvic and hindlimb kinematics between species, particularly those occupying different habitats. These different swimming strategies employed by various species of frogs along with our finding that AQ taxa exhibit the most variation in ratios suggest swimming is a less functionally (and morphologically) constrained type of locomotion than jumping, although future functional studies are needed to test such a hypothesis explicitly.

### 4.2 Skeletal pelvic morphology should be considered along a continuum

Both the pPCA and ratio spectrum analyses support the findings of previous studies in that sacral expansion is the key driver of morphological variation and the primary determinant of locomotor mode in frogs (Emerson, 1979; 1982; Jorgensen & Reilly, 2013; Buttimer et al., 2020). A narrow ESD and a low ESD/hindlimb length ratio are associated with TJ, whereas high values of the same ratio are associated with AQ, supporting our sixth hypothesis (H6). Sacral width, pelvis length and iliac angle are also expected to vary with locomotor mode. BWH and WH have a wider and longer pelvis (Table 2) to enable lateral rotation (Emerson, 1982), as it creates room for larger muscles and the potential for longer external moment arms about the iliosacral joint. In our study, AJ and TJ have the narrowest sacral width while BWH had the widest (Jorgensen & Reilly, 2013; Simons, 2008). Furthermore, the iliac angle was smallest for TJ and largest for WH (Table 2).

**FIGURE 6:**
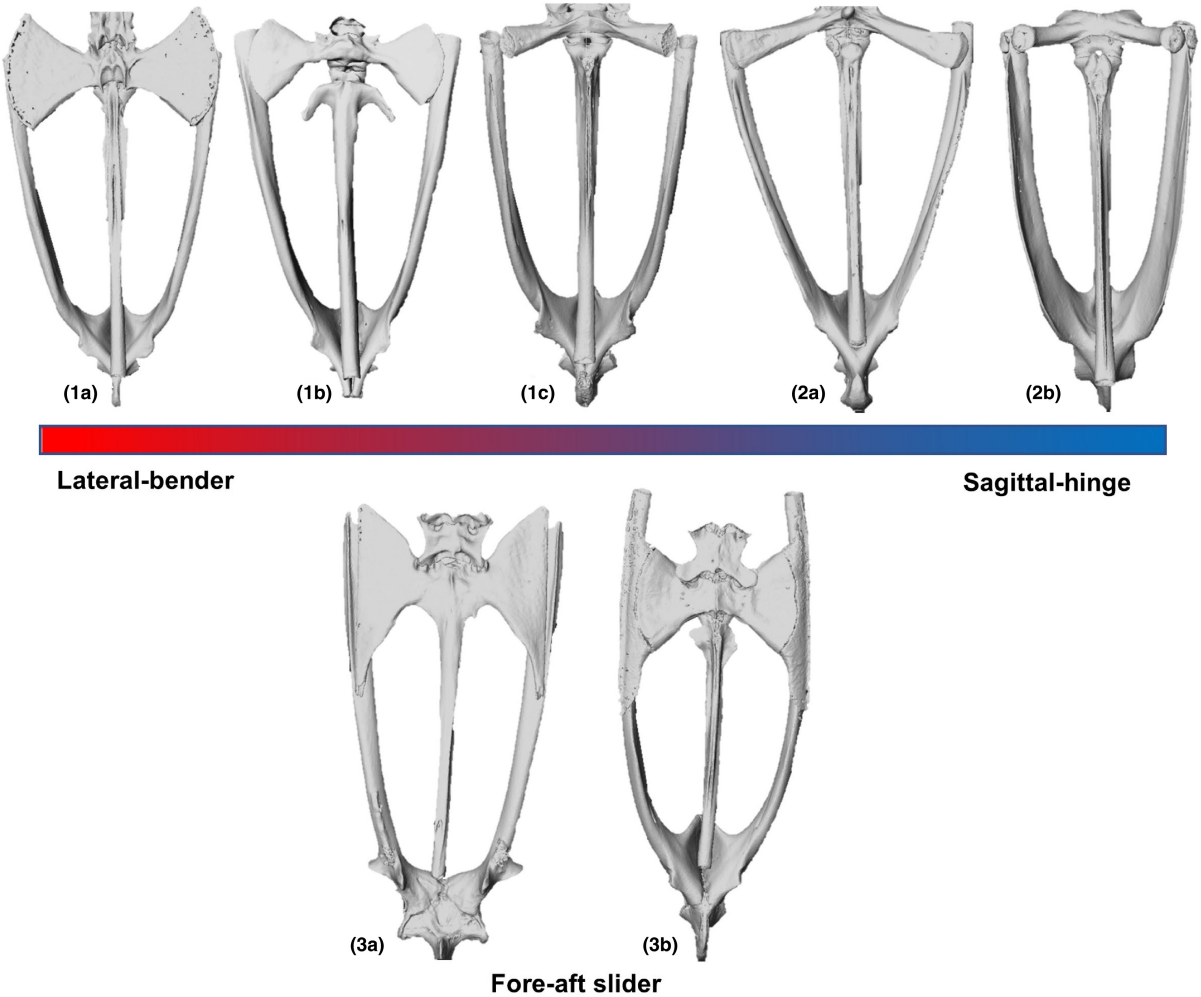
Three distinct pelvis types following the work of Reilly and Jorgensen (2011) and Emerson (1979; 1982). Lateral‐bender and sagittal‐hinge morphs appear along a morphological continuum (coloured bar). (1a) *Ansonia mcgreggori*—WH, Hyloidea; (1b) *Alytes obstetricians*—BWH, basal; (1c) *Batrachyla taeniata*—WH, Hyloidea; (2a) *Hemiphractus proboscideus*—AJ, Hyloidea; (2b) *Ptychadena oxyrhynchus*—riparian TJ, Ranoidea; (3a) *Xenopus calcaratus* – AQ, basal; (3b) *Callulina kisiwamsitu*—BWH, Ranoidea.

Walker‐hoppers, swimmers, and jumpers have been associated with ‘lateral‐bending’, ‘fore‐aft sliding’ and ‘sagittal‐hinge’ pelvis types, respectively (Emerson, 1979, 1982; Reilly & Jorgensen, 2011). Jorgensen and Reilly (2013) found that all except one species of TJ in their study possess a sagittal‐hinge pelvis. However, the record for best jumping performance (the equivalent of 55.2 times body length) is currently held by the pelodryadinine hylid *Litoria nasuta* (James & Wilson, 2008), which has a large sacral expansion, atypical for jumping species. Computational simulations suggest that a sagittal‐hinge mechanism is not obligatory for jumping as it is mostly used to fine‐tune jump trajectory (Richards et al., 2018). Frogs could hypothetically attain greater jumping performance through extreme elongation of the hindlimbs to compensate for the lack of a sagittal‐hinge pelvis type. Additionally, species with the sagittal‐hinge pelvic morphology which attains the theoretical optimum for jumping may only be able to improve jumping performance by further elongating their hindlimbs. The present study initially aimed to explore relationships among pelvic morphology and locomotor mode in more detail, but locomotor categories did not neatly align with Emerson's pelvic types, an observation also made by Simons (2008) and Soliz et al. (2017). In particular, lateral‐bending and sagittal‐hinge pelvis types appear to blend along a morphological continuum, especially in the shape of the sacral diapophyses (Figure 6; 1a–2b), suggesting more complex links between form and function in anuran pelvic structures than previously thought. For example, the sacral shape in *Batrachyla taeniata* differs significantly from that of *Ansonia mcgregori*, both of which are walker‐hopper hyloids classed as having a lateral‐bending pelvis type. Sacral shape in *B. taeniata* appears more similar to that of *Ptychadena oxyrhynchus*, which has a sagittal‐hinge pelvis type according to Reilly and Jorgensen (2011). In comparison, FA pelvic types appear relatively consistent in shape (Figure 6; 3a–3b). These observations support our conclusion that multiple anatomical solutions are potentially available to achieve particular locomotor styles and functional performance or access particular habitats.

One important limitation of our study that should be noted is that microCT scanning of unstained specimens, such as those used exclusively in the present study to capture skeletal shape, typically do not permit visualization of lower density connective tissues, as all soft tissues (muscles, cartilage, tendons, etc.) typically present with the same density/grayscale value in the data and cannot be distinguished from each other. Thus, poorly mineralised cartilaginous and ligamentous structures that form an integral part of the sacro‐iliac joint (Emerson, 1979; Manzano & Barg, 2005; Reilly & Jorgensen, 2011) may not be clear in our data and subtle but important anatomical differences between taxa may be missed.

### 4.3 Locomotor mode has the greatest impact on morphology

Several ecomorphometric studies suggest that the frog body plan enables responses to a broad array of mechanical challenges and environmental uncertainty, and therefore allows them access to a variety of locomotor styles and habitats (Nauwelaerts et al., 2007; Gomes et al., 2009; Moen et al., 2013; Vidal‐Garcia et al., 2014; Tulli et al., 2016; Soliz et al., 2017). Though this generalised morphology could represent a morphological optimum that is constrained by phylogenetic inertia (Soliz et al., 2017), strong correlations have been found between performance, morphology, and microhabitat, regardless of phylogeny or geographical location (Gomes et al., 2009; Moen et al., 2013). Our results indicate that similar morphological structures and locomotor modes occur across unrelated taxa, in particular for TJ and AJ, suggesting that locomotor mode is a more important driver of morphological evolution than phylogeny (Figure 2; Table S4; Emerson, 1988; Reilly & Jorgensen, 2011). Habitat type is a weaker driver of morphological evolution (Table 1), demonstrated by the disappearance of locomotor trends when grouping by habitat type. For example, BWH have the most robust forelimb and hindlimb, but this is hidden when grouped by habitat as TJ, WH and BWH have contrasting morphologies. When grouped by habitat, aquatic taxa appear to have the most robust forelimb and hindlimb (Table 2). However, this is not to say that habitat type plays no role in shaping morphology. For example, the terrestrial group has a high level of accuracy in both the LDA (88.5%; Table 3) and pFDA (83.3%; Table 4), indicating that common functional requirements and constraints involved with living a terrestrial lifestyle, such as greater weight‐bearing compared to arboreal and aquatic taxa, could result in a predictably similar morphology. In summary, future studies should be cautious, as using broad habitat types alone is not sufficient for explaining morphological variation in anurans.

### 4.4 Skeletal morphology can be a powerful predictor of function and ecology

Predictive models yielded strikingly similar results, regardless of whether they incorporated phylogenetic history (see supplementary dataset). Almost every species had the same group predicted in both the pFDA and LDA for their locomotor mode (96.3%), habitat type (97%) and phylogenetic group (94.5%). Although there were some cases where predictions differed to the locomotor (41.5%), habitat (35.4%) or phylogenetic (16.5%) group allocated in our dataset, several of these ‘misclassifications’ were when the alternative locomotor mode was correctly predicted instead.

The predictive models performed differently when we analysed the 10 neobatrachian species of unknown phylogenetic group. As Neobatrachia is comprised of Ranoidea and Hyloidea, these unclassified taxa were expected to be predicted as belonging to one of these groups, which was the outcome of the LDA. The pFDA yielded an unexpected result in that each of these taxa was categorised as basal according to their skeletal morphology. In the Jetz and Pyron (2018) tree, these species occupy a basal position outside of Hyloidea or Ranoidea, indicating that the specialised morphologies that distinguish the more derived members of those groups have not yet evolved. Figure 2.2 demonstrates how neobatrachians are central within phylogenetic group morphospace. Their phylogenetic uncertainty implies that these species have always been difficult to classify based on their skeletal morphology alone, so the pFDA may be reflecting this. Overall, the results of our predictive analyses provide confidence that we could extend our inquiry to extinct taxa and predict unknown locomotor modes using measurements from fossils (Gómez & Lires, 2019; Lires et al., 2016).

### 4.5 Riparian morphology is influenced more by jumping than swimming

Previous studies have argued that swimming and jumping are morphologically and functionally similar (Buttimer et al., 2020; Lires et al., 2016; Nauwelaerts et al., 2007; Vidal‐García et al., 2014), a finding not reflected by our analyses. Incorporating a semi‐aquatic habitat type permitted useful insight into the role of locomotor mode in determining morphology and suggests why our results differ from the cited studies. Even though riparian species spend approximately half their time in an aquatic environment, their skeletal measurements indicate morphology that is more suited to jumping than swimming (Figure 2(b)). Similar to TJ, riparian taxa have the smallest ESD, longest tibiofibula relative to femur, and the longest hindlimb relative to forelimb. They also have the lowest ratio of femur to total hindlimb length, while aquatic frogs have the highest. The PERMANOVA (Table S4) shows that riparian and aquatic taxa are significantly different from each other and that there is no significant separation between terrestrial and riparian taxa. Riparian species were most often mistaken for inhabiting terrestrial environments, according to both our LDA (58.1%; Table 3) and pFDA (64.5%; Table 4), even though terrestrial taxa have the most frequently correct classifications (LDA 88.5%; pFDA 83.3%). Additionally, in terms of locomotor mode, TJ were never misclassified as AQ, contrasting previous findings (Lires et al., 2016). These results all suggest that riparian skeletal morphology may be less strongly influenced by the functional demands for swimming than jumping performance. Indeed, data on jumping performance in frogs combined with ancestral state reconstructions suggest that the evolution of high jumping performance appears to be correlated with the evolution of elongated hind limbs within Neobatrachia (Herrel et al., 2016).

Despite these significant differences in morphology, there is unlikely to be a performance trade‐off between the two locomotor modes (Herrel et al., 2014; Nauwelaerts et al., 2007; Soliz et al., 2017). Several characteristics have previously been suggested to be advantageous for jumping as well as for swimming. For example, riparian taxa in our sample have the longest feet, potentially to increase the surface area of the ‘paddle’ for underwater propulsion, and for generating a larger force during jumping. Furthermore, as previously noted, studies of swimming kinematics across frog species have demonstrated that taxa with different ecologies may employ different swimming strategies, potentially reflecting differences in morphology (Richards, 2010; Robovska‐Havelkova et al., 2014). Overall, these patterns permit insightful inference into the activity of specific sets of muscles and their major roles in each locomotor style.

### 4.6 Assigning locomotor and habitat type categories

Grouping each species under a discrete locomotor mode and habitat type to perform comparative analyses, though common practice in the literature (e.g., Jorgensen & Reilly, 2013), is not straightforward. For example, burrowing locomotion can be further subdivided into taxa with burrow using their forelimbs or hindlimbs, but this data is not readily available for many taxa (Keeffe & Blackburn, 2020). Conflicting observations between field biologists can also undermine analytical power (pers. comms). Previous studies have attempted to account for this by examining locomotor modes and habitat type as relative proportions (e.g., Soliz et al., 2017). We have incorporated some level of variability by including potential alternative locomotor modes and habitats. However, there is a need for more detailed and publicly accessible accounts of animal behaviour and habitat usage and in‐depth sensitivity tests of the effect of these categorisations.

### 4.7 Conclusions and future research

Our results support most of our hypotheses about trends in skeletal morphology and their power to predict locomotor style and habitat. How our findings translate to changes in muscular morphology, and what the biomechanical implications of these changes are, remain largely unknown. Indeed, it has been demonstrated both in anurans and more widely across jumping animals that muscle size and mechanics have a strong impact on jumping performance (James et al., 2007). Functional analyses are an essential next step to establish how variable morphology directly impacts locomotion in frogs (e.g., Richards & Porro, 2018). By segmenting frog skeletons from CT scans for use in morphometrics analyses, we have contributed towards producing a library of 3D digital models that can be used in future biomechanical analyses of locomotor function (Richards, 2019), such as the extent to which differing hindlimb proportions directly impact hindlimb motion and locomotor performance.

We found sacral expansion to be the strongest predictor of locomotor function, as also uncovered in other studies (Jorgensen & Reilly, 2013; Petrović et al., 2017; but see Lires et al., 2016). Previous studies have suggested that pelvic characteristics may be best considered as a continuum since the structures defining Emerson's (1979) three pelvic types are notoriously difficult to classify consistently and reliably across the frog phylogeny (Simons, 2008; Soliz et al., 2017; Tulli et al., 2016). This study presents novel evidence supporting this proposal across a broad range of anuran families, locomotor modes and habitat types (Figure 6). We will not know the significance of pelvic morphology until more functional studies are done which consider the pelvis‐hindlimb as a whole unit. Additionally, while we could determine the presence of dorsal ridges on the ilia and the urostyle, as well as the shape of the sacral diapophyses, with relative ease, investigating sacral‐urostylic articulation proved difficult without contrast‐enhanced CT scans. Swimmers and burrowers are hypothesized to have evolved a fused urostyle to limit lateral bending and create greater force through the hindlimbs, while a bicondylar sacro‐urostylic junction may play a similar role in jumping frogs (Pugener & Maglia, 2009; Jorgensen & Reilly, 2013). A future application of contrast‐enhanced CT scans to specifically investigate the sacral‐urostylic articulation would provide further insight into the importance of pelvic morphology in locomotion.

Our predictive analyses demonstrated how pelvic type, locomotor mode and habitat type can vary even at the species‐level, so entire families should not be allocated to one group, a common practice in previous studies (Jorgensen & Reilly, 2013; Reilly & Jorgensen, 2011). Which families are backed into an evolutionary corner of morphological design, and which show true diversity? Are more diverse groups experiencing a lack of evolutionary constraints, or are there multiple anatomical solutions to achieving the same locomotor style? Many large families, such as the Hylidae, demonstrate large levels of variation, while other families are more conservative (Moen et al., 2013; Soliz et al., 2017; Vidal‐García et al., 2014). The high anatomical variation we have observed at the species‐level indicates varied and complex locomotor functions, suggesting that diversity within families is overlooked and underappreciated.

## AUTHOR CONTRIBUTIONS

L. B. P. and C. T. R. conceived and supervised the study. A. L. collected all of the data and, with assistance from L. B. P. and M. R., performed the data analysis. A. L. drafted the manuscript, and all authors provided constructive feedback.

## CONFLICT OF INTEREST STATEMENT

The authors declare no conflict of interest.

### OPEN RESEARCH BADGES

This article has earned an Open Data badge for making publicly available the digitally‐shareable data necessary to reproduce the reported results. The data is available at [[insert provided URL from Open Research Disclosure Form]].

## Supporting information


Data S1.

Data S2.

Data S3.

Data S4.

**Data S5.** Supporting information

## Data Availability

The data that support the findings of this study are all included in the supporting information.
