# Supplementary material for: Locomotor, ecological and phylogenetic drivers of skeletal proportions in frogs
Source: J Anat. 2023 May 19;243(3):404–20. doi: 10.1111/joa.13886 (PMC10439368; doi:10.1111/joa.13886)
Supplement: Supplementary file 3 — Data S3. [file JOA-243-404-s003.docx]

**Abbreviations**

LM – locomotor mode:

- AJ (Arboreal Jumper)
- TJ (Terrestrial Jumper)
- WH (Walker-Hopper)
- BWH (Burrower-Walker-Hopper)
- AQ (Swimmer)

HT – habitat type

- Aquatic – spends the majority of its time in water.
- Arboreal – spends the majority of its time in vegetation/trees.
- Terrestrial – spends the majority of its time on the ground.
- Riparian – spends approximately an equal amount of time in water as it does in terrestrial environments, i.e., relies on water outside of just for the purposes of reproduction.

Pelvic design:

- Sacrum shape is based on the descriptions of Emerson's (1979) three pelvic types – LB (lateral bending); FA (fore-aft sliding); SH (sagittal-hinge).
- Iliac crest
  - smooth (may have dorsal crest that extends no further than one-third of the way down the ilium)
  - ridge (the ilium must have a crest that extends all the way down the length of the ilium, tapering off at the end)
- Urostylic crest
  - smooth (may have a dorsal crest that extends no further than one-third of the way down the urostyle – remnant of the neural arch)
  - half (crest extends no further than halfway down the length of the urostyle)
  - ridge (the urostyle must have a crest that extends all the way down the length of the urostyle, occasionally tapering off at the end)
  - lateral (crest expands laterally)
  - T-shaped (crest forms the shape of a 'T' at the most proximal end of the urostyle).

‘**Full dataset’**

This data contains all of the raw measurement and categorical data acquired during the course of this project.

Measurement data and pelvic features were collected using micro-CT scans that are available on MorphoSource.org. All ARK identifiers can be found under ‘Scan source’.

Locomotor mode & habitat type data - determined by reading relevant literature, accessing AmphibiaWeb and the IUCN website, and personal communications with Andrew Gray (University of Manchester) and Dave Blackburn (University of Florida). See ‘LM_citation’ and ‘habitat_citation’.

Phylogenetic clade - this categorisation was based on the placement of the study taxa in the Jetz & Pyron (2017) phylogeny.

Note that the variable ‘gap’ (the space between the base of the skull and the anterior end of the vertebral column) was measured to calculate snout-vent length (see below) and is not used in analyses.

* denotes that the pelvis features which differ to previous findings in Reilly & Jorgensen (2011) and Jorgensen & Reilly (2013).

‘**Structural dataset’**

This dataset is the same as the full dataset, except that some measurements have been combined to form larger structural measurements of frog morphology:

- Snout-vent length (SVL): skull + gap + vertebrae + pelvis lengths
- Hindlimb length: femur + tibiofibula + calcaneus + foot lengths
- Forelimb length: humerus + radioulna + hand lengths

**‘Predictive analyses dataset’**

This dataset contains details of the potential secondary locomotor modes and habitat types tested in predictive models, and their sources. It also contains the results of the linear discriminant analyses (LDA) and phylogenetic flexible discriminant analyses (pFDA) for locomotor mode, habitat type and phylogenetic group.

* - the alternative option was correctly predicted
