# Supplementary material for: Locomotor, ecological and phylogenetic drivers of skeletal proportions in frogs
Source: J Anat. 2023 May 19;243(3):404–20. doi: 10.1111/joa.13886 (PMC10439368; doi:10.1111/joa.13886)
Supplement: Supplementary file 5 — Data S5. Supporting information [file JOA-243-404-s005.docx]

**SUPPLEMENTARY INFORMATION**

**Table S1 –** Descriptions of our skeletal measurements with their abbreviations.

|  | **Measurement** | **Description** |
| --- | --- | --- |
| **Body measurements** | Snout-vent length [SVL] | Skull + Gap + Vertebrae + Pelvis lengths |
|  | Skull length | The most anterior point of premaxilla to the base of the skull along central line. |
|  | Gap between skull and vertebrae | Sometimes required for measuring snout-vent length. The space between the base of the skull to the most cranial point of the vertebral column along the central line. |
|  | Vertebral length [vert] | The most cranial to most caudal point (before the sacral vertebrae attach to vertebral body) of the vertebral column along the central line. When curved (or in one case, broken), this was measured in two parts. |
| **Pelvic measurements** | Pelvis length [pelv] | The most anterior point of where the sacral vertebrae attach to the vertebral column to the most posterior end of the pelvis/ischium. |
|  | Sacral width [sacr_w] | The maximal ossified width of the sacral vertebrae at the widest point. Sesamoids and cartilaginous caps were not included. |
|  | Expansion of the sacral diapophyses [ESD] | The greatest length between the cranial and caudal edges of the left sacral diapophysis adjacent to the vertebral centrum. Cartilaginous caps were not included. |
|  | Ilium length [ilium] | The distance from the anterior tip of the left ilium to the lateral process of the ilium. |
|  | Anterior iliac distance (1) | The distance between the anterior end of each ilium. |
|  | Posterior iliac distance (2) | The distance between the lateral process of each ilium. |
|  | Urostylic length [uro] | The most anterior to most posterior end of the urostyle. When the urostyle is fused to the sacral vertebrae, the measurement is from the most posterior/caudal side of sacral vertebrae to the end of the urostyle. |
| **Hindlimb measurements** | Hindlimb length [HL] | Femur + Tibiofibula + Foot |
|  | Femur length [fem] | The maximum ossified length measured from proximal femoral head to the distal end. |
|  | Femur width [fem_w] | The maximum ossified width measured in the midpart of the femur. |
|  | Tibiofibula length [tib] | The maximum ossified length measured from the proximal end of the tibiofibula to the distal end. |
|  | Calcaneus length [calc] | The maximum ossified length measured from the proximal end of the calcaneus to the distal end. |
|  | Foot total length [foot] | Heel + Metatarsal + Toe total |
|  | Heel | The distance between the most distal calcaneus point to the most proximal end of the metatarsal (includes all the bones ‘floating about’ in the middle). If there is no gap between calcaneus and metatarsal, the measurement is zero. |
|  | Metatarsal | The most proximal to most distal end of the metatarsal of the longest toe (usually the fourth). |
|  | Toe total | The most distal end of the metatarsal to the tip of the longest toe (usually the fourth). There were usually four bones in each toe. Individual measurements were taken along each bone, from the distal end of last measurement to the next bone, so including the gap prior to that bone. Sometimes the last bone in a finger or toe has been dislocated. In this case, the gap between the last and penultimate bone was not measured, just the length of the bone itself. |
| **Forelimb measurements** | Forelimb length [FL] | Humerus + Radio-ulna + Hand total |
|  | Humerus length [hum] | The maximum ossified length of the humerus measured from the proximal end to the most distal end. |
|  | Humerus width [hum_w] | The maximum ossified width measured in the midpart of the humerus. |
|  | Radio-ulna length [rad] | The maximum ossified length of the radio-ulna measured from the proximal end to the most distal end. |
|  | Hand total [hand] | Wrist + Fingers total |
|  | Wrist | The distance between the most distal radio-ulna measurement to the most proximal end of the first bone of the longest finger (includes all the bones ‘floating about’ in the middle). |
|  | Fingers total | The most proximal end of the first bone of the longest finger to the tip (usually the third). There were usually four bones in each finger. Individual measurements were taken along each bone, from the distal end of last measurement to the next bone, so including the gap prior to that bone. |

**Figure S1 –** Method for Iliac angle calculation. Ilium length is the hypothenuse, and the opposite is half of anterior iliac distance (1) minus half of the posterior iliac distance (2). Arcsine (opposite/hypothenuse) was used to get the angle (a) the ilia diverge from a line parallel to the midline.


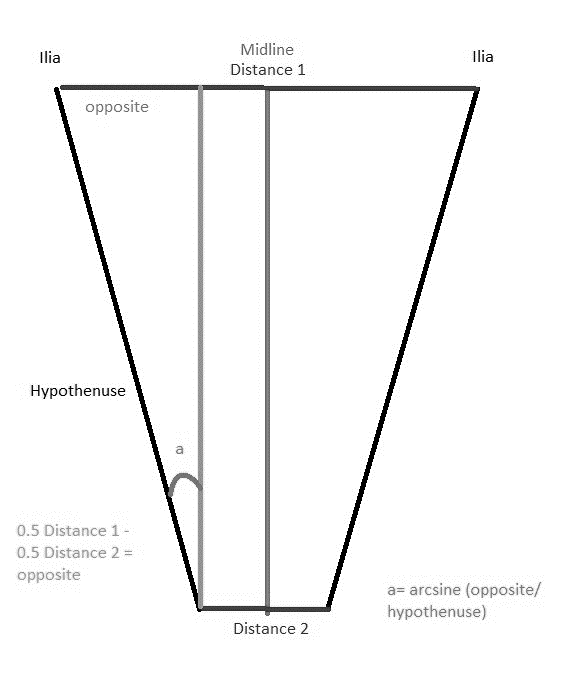


Urostyle

**Table S2 –** Substitute taxa used in our phylogeny. A * represents where a species name has been updated.

| **This study** | **Jetz and Pyron (2017)** | **Reference** |
| --- | --- | --- |
| *Amnirana albolabris* | *Hylarana albolabris ** | IUCN (2020) |
| *Amnirana galamensis* | *Hylarana galamensis ** | IUCN (2020) |
| *Cornufer guppyi* | *Discodeles guppyi ** | IUCN (2020) |
| *Cornufer guentheri* | *Ceratobatrachus guentheri ** | IUCN (2020) |
| *Boana boans* | *Hypsiboas boans ** | IUCN (2020) |
| *Kalophrynus sinensis* | *Kalophrynus pleurostigma* | No study found including this particular species – chose *K. pleurostigma* as an estimate for branch lengths. |
| *Lithobates vibicarius* | *Rana vibicaria ** | IUCN (2020) |
| *Micrixalus adonis* | *Micrixalus fuscus* | Biju *et al.* (2014) |
| *Niceforonia araiodactyla* | *Hypodactylus araiodactylus ** | IUCN (2020) |
| *Phlyctimantis maculatus* | *Kassina maculata ** | Portik & Blackburn (2016) |
| *Sclerophrys dodsoni* | *Duttaphrynus dodsoni ** | IUCN (2020) |
| *Triprion spinosus* | *Anotheca spinosa ** | IUCN (2020) |
| *Walkerana phrynoderma* | *Indirana phrynoderma ** | Dahanukar *et al.* (2016) |
| *Xenopus calcaratus* | *Xenopus* *epitropicalis* | Evans *et al.* (2015) |

**Table S3** – pPCA loadings for the full dataset from the first four axes. For each PC axis, light and dark boxes highlight the largest positive and negative loadings respectively. Abbreviations can be found in Table S1.

| Axis | PC1 | PC2 | PC3 | PC4 |
| --- | --- | --- | --- | --- |
| Total variance explained (%) | 34.23 | 21.87 | 9.76 | 7.75 |
| skull | -0.388 | -0.007 | -0.421 | 0.122 |
| vert | 0.327 | -0.139 | -0.448 | 0.413 |
| pelv | 0.031 | -0.199 | -0.236 | 0.077 |
| ESD | 0.964 | 0.198 | 0.140 | 0.042 |
| sacr_w | 0.316 | -0.090 | -0.150 | -0.345 |
| ilium | 0.322 | -0.009 | -0.091 | 0.140 |
| uro | -0.131 | 0.129 | -0.063 | -0.157 |
| fem | -0.611 | 0.475 | 0.411 | -0.200 |
| fem_w | -0.035 | -0.673 | 0.348 | 0.189 |
| tib | -0.609 | 0.565 | 0.440 | -0.209 |
| calc | -0.357 | 0.738 | 0.190 | -0.342 |
| foot | -0.512 | -0.144 | 0.474 | 0.563 |
| hum | -0.017 | -0.018 | -0.652 | -0.264 |
| hum_w | 0.043 | -0.894 | 0.102 | -0.317 |
| rad | -0.021 | 0.255 | -0.668 | -0.261 |
| hand | -0.388 | 0.162 | -0.331 | 0.496 |

**Table S4 -** Results from the pairwise PERMANOVA analyses of the full dataset, which test for statistical differences between the means of locomotor modes, habitat types and phylogenetic clades for 164 frog taxa.

| **Pairwise comparison** | **R^2^ *_adonis_*** | **P *_adonis_*** | **P *_adonis adjusted_*** |
| --- | --- | --- | --- |
| **Locomotor mode** | | | |
| AJ vs TJ | 0.147 | 0.0001 | **0.001** |
| AJ vs WH | 0.158 | 0.0001 | **0.001** |
| AJ vs BWH | 0.272 | 0.0001 | **0.001** |
| AJ vs AQ | 0.196 | 0.0004 | **0.004** |
| TJ vs WH | 0.178 | 0.0001 | **0.001** |
| TJ vs BWH | 0.327 | 0.0001 | **0.001** |
| TJ vs AQ | 0.195 | 0.0001 | **0.001** |
| WH vs BWH | 0.054 | 0.0218 | 0.218 |
| WH vs AQ | 0.055 | 0.1230 | 1.000 |
| BWH vs AQ | 0.014 | 0.6667 | 1.000 |
| **Habitat type** | | | |
| Arboreal vs Terrestrial | 0.065 | 0.0001 | **0.0006** |
| Arboreal vs Riparian | 0.131 | 0.0001 | **0.0006** |
| Arboreal vs Aquatic | 0.201 | 0.0005 | **0.0030** |
| Terrestrial vs Riparian | 0.042 | 0.0044 | 0.0264 |
| Terrestrial vs Aquatic | 0.031 | 0.0295 | 0.1770 |
| Riparian vs Aquatic | 0.198 | 0.0006 | **0.0036** |
| **Phylogenetic clade** | | | |
| Ranoidea vs Hyloidea | 0.020 | 0.0433 | 0.2598 |
| Ranoidea vs Basal | 0.238 | 0.0001 | **0.0006** |
| Ranoidea vs Neobatrachia | 0.041 | 0.0299 | 0.1794 |
| Hyloidea vs Basal | 0.193 | 0.0001 | **0.0006** |
| Hyloidea vs Neobatrachia | 0.325 | 0.0363 | 0.2178 |
| Basal vs Neobatrachia | 0.155 | 00073 | 0.0438 |

**Table S5** – Shape PCA loadings for the structural dataset from the first four axes. For each PC axis, blue and red boxes highlight the largest positive and negative loadings respectively. Abbreviations can be found in Table S1.

| Axis | PC1 | PC2 | PC3 | PC4 |
| --- | --- | --- | --- | --- |
| Total variance explained (%) | 66.9 | 16.8 | 5.1 | 3.8 |
| SVL | -0.144 | -0.096 | 0.004 | -0.161 |
| ESD | 0.908 | -0.151 | 0.019 | 0.104 |
| sacr_w | -0.015 | -0.037 | 0.157 | -0.694 |
| ilium | -0.004 | -0.139 | -0.111 | 0.135 |
| uro | -0.157 | -0.230 | -0.168 | 0.197 |
| HL | -0.289 | -0.344 | -0.096 | 0.476 |
| fem_w | -0.116 | 0.343 | 0.821 | 0.229 |
| FL | -0.183 | -0.145 | -0.148 | -0.372 |
| hum_w | 0.0004 | 0.798 | -0.478 | 0.087 |

**Table S6** – Shape PCA loadings for the full dataset from the first four axes. For each PC axis, blue and red boxes highlight the largest positive and negative loadings respectively. Abbreviations can be found in Table S1.

| Axis | PC1 | PC2 | PC3 | PC4 |
| --- | --- | --- | --- | --- |
| Total variance explained (%) | 54.4 | 17.3 | 6.0 | 4.2 |
| skull | -0.173 | 0.060 | 0.146 | -0.149 |
| vert | 0.060 | 0.014 | 0.300 | -0.298 |
| pelv | 0.000 | 0.057 | -0.001 | -0.145 |
| ESD | 0.864 | -0.314 | -0.143 | -0.006 |
| sacr_w | 0.081 | 0.049 | 0.201 | 0.099 |
| ilium | 0.087 | -0.012 | -0.039 | -0.168 |
| uro | -0.051 | -0.054 | -0.069 | -0.074 |
| fem | -0.176 | -0.183 | -0.180 | 0.150 |
| fem_w | 0.018 | 0.392 | -0.214 | -0.280 |
| tib | -0.252 | -0.301 | -0.295 | 0.242 |
| calc | -0.180 | -0.414 | -0.060 | 0.371 |
| foot | -0.181 | 0.074 | -0.481 | -0.324 |
| hum | -0.057 | 0.059 | 0.391 | 0.102 |
| hum_w | 0.140 | 0.651 | -0.171 | 0.566 |
| rad | -0.042 | 0.004 | 0.467 | 0.166 |
| hand | -0.137 | -0.083 | 0.148 | -0.252 |


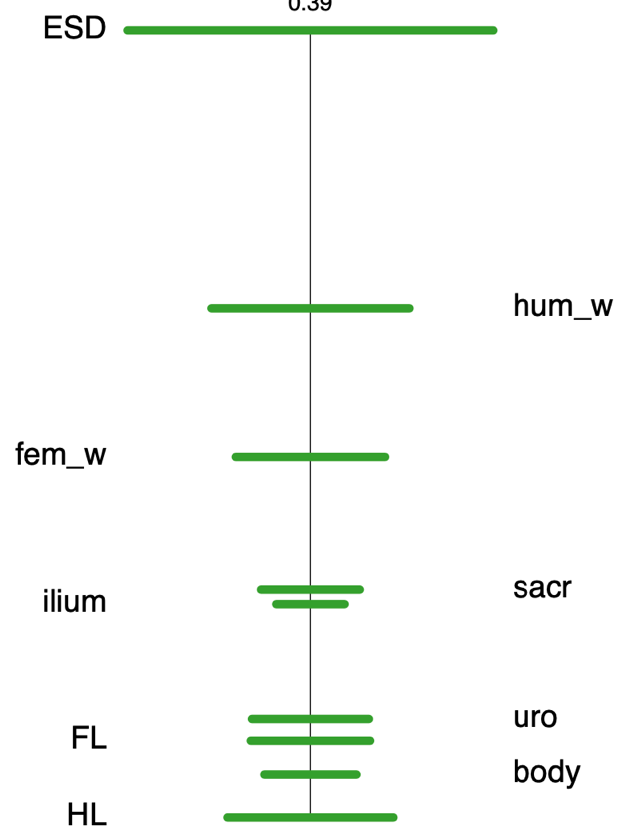

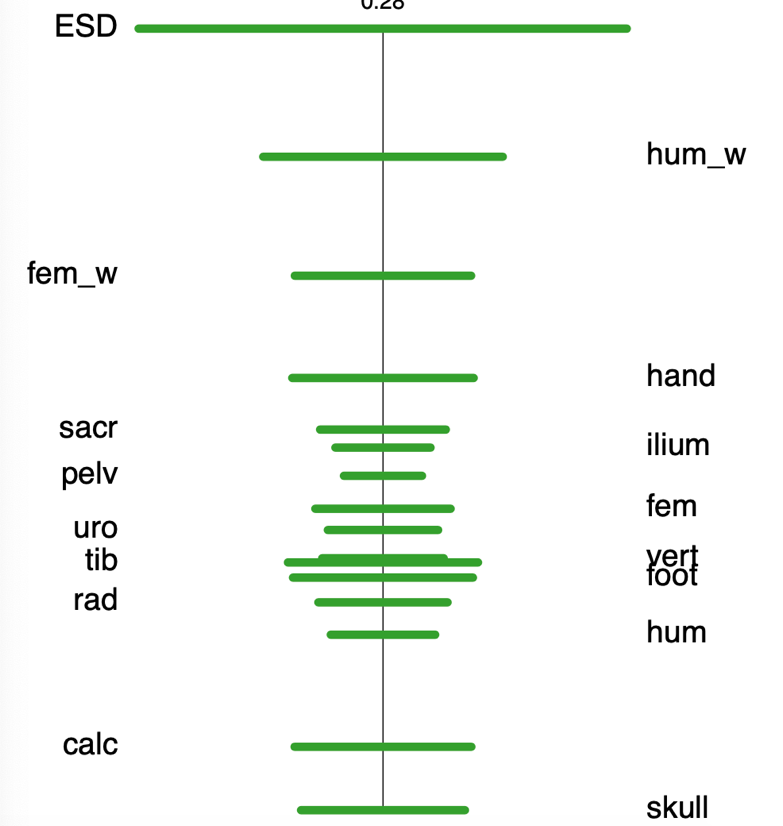


**b)**

0.28

0.23

**a)**

0.39

0.3

SVL

**Figure S2 –** Allometry ratio spectrum (Baur & Leuenberger, 2011) for a) our structural dataset containing total body and limb lengths and b) our main dataset. The bars represent 68% confidence intervals based on 999 bootstrap replicates. As they are wide, the error bars suggest there is little allometric variation in the data.

**Table S7 –** Phylogenetic signal (λ), residual standard error (R) and Akaike Information Criterion (AIC) for each PGLS model of PC1 and PC2 (full dataset). The best model is highlighted in bold.

| **Model** | **λ** | **R** | **AIC** |
| --- | --- | --- | --- |
| **PC1~LM+hab** | 1.024 | 0.225 | **-143.94** |
| PC1~LM | 1.039 | 0.232 | -141.79 |
| PC1~hab | 1.050 | 0.241 | -132.46 |
| PC1~1 | 0.000 | 0.231 | -133.32 |
| PC2~LM+hab | 0.638 | 0.130 | -247.70 |
| **PC2~LM** | 0.650 | 0.131 | **-248.02** |
| PC2~hab | 0.800 | 0.151 | -224.35 |
| PC2~1 | 0.000 | 0.185 | -207.09 |

**References**

Baur, H. and Leuenberger, C. (2011). Analysis of ratios in multivariate morphometry. Systematic Biology, 60(6), 813-825.

Biju, S.D., Garg, S., Gururaja, K.V., Shouche, Y. and Walujkar, S.A., 2014. DNA barcoding reveals unprecedented diversity in Dancing Frogs of India (Micrixalidae, *Micrixalus*): a taxonomic revision with description of 14 new species. *Ceylon Journal of Science (Biological Sciences)*, *43*(1), pp.37-123.

Dahanukar, N., Modak, N., Krutha, K., Nameer, P.O., Padhye, A.D. and Molur, S., 2016. Leaping frogs (Anura: Ranixalidae) of the Western Ghats of India: An integrated taxonomic review. *Journal of Threatened Taxa*, *8*(10), pp.9221-9288.

Evans, B.J., Carter, T.F., Greenbaum, E., Gvoždík, V., Kelley, D.B., McLaughlin, P.J., Pauwels, O.S., Portik, D.M., Stanley, E.L., Tinsley, R.C. and Tobias, M.L., 2015. Genetics, morphology, advertisement calls, and historical records distinguish six new polyploid species of African clawed frog (*Xenopus*, Pipidae) from West and Central Africa. *PLoS One*, *10*(12).

Jetz, W. and Pyron, R.A., 2018. The interplay of past diversification and evolutionary isolation with present imperilment across the amphibian tree of life. *Nature Ecology & Evolution*, *2*(5), pp.850-858.

Portik, D.M. and Blackburn, D.C., 2016. The evolution of reproductive diversity in Afrobatrachia: A phylogenetic comparative analysis of an extensive radiation of African frogs. *Evolution*, *70*(9), pp.2017-2032.
